# A ‘traumatic’ mechanical small bowel obstruction after blunt pelvic trauma

**DOI:** 10.1093/jscr/rjad722

**Published:** 2024-02-19

**Authors:** Sarah Kecman, Sebastian Schindera, Mark Hartel, Alexander Gräfitsch

**Affiliations:** Department of Surgery, Kantonspital Aarau, Tellstrasse 25, Aarau 5001, Switzerland; Department of Radiology, Kantonspital Aarau, Tellstrasse 25, Aarau 5001, Switzerland; Department of Surgery, Kantonspital Aarau, Tellstrasse 25, Aarau 5001, Switzerland; Department of Surgery, Kantonspital Aarau, Tellstrasse 25, Aarau 5001, Switzerland

**Keywords:** traumatic abdominal wall hernia, acetabular fracture, bowel obstruction

## Abstract

Traumatic abdominal wall hernia (TAWH) is a rare form of herniation caused by blunt trauma that can lead to intestinal obstruction. This report details a rare case of delayed mechanical ileus resulting from TAWH due to an acetabular fracture. The patient was successfully treated with laparoscopic closure of the peritoneal orifice, followed by orthopaedic repair of the fracture. The presented scenario underlines the importance of timely diagnosis and interdisciplinary collaboration in addressing complex TAWH cases.

## Introduction

Traumatic abdominal wall hernia (TAWH) can occur after blunt traumatic abdominal wall disruption [1]. First described in 1906 [2], it remains a rare injury with an estimated prevalence of 1%; consequently, neither a classification nor a diagnostic pathway have been established. Notably, TAWH due to pelvic fractures are even rarer, with only 20 cases reported in the literature, from which 7 were associated with an acetabular fracture [3]. Some cases of delayed bowel obstruction caused by a TAWH have already been reported [4, 5]. This case report details an occurrence of a mechanical ileus arising from an acetabular fracture. The distinctive aspect lies in the treatment approach, wherein laparoscopic surgery was performed before the open reduction and fixation of the fracture.

## Case presentation

An 83-year-old woman with a history of untreated osteoporosis was admitted to the emergency department of a community hospital after tripping in a supermarket. She reported immobilizing pain in the right hemipelvis and tenderness over the right inguinal region. The ATLS primary survey did not reveal any additional injuries. An X-ray of the pelvis identified a right two-column fracture of the acetabulum, confirmed by a native CT-scan of the abdomen. Despite a White cell count (WCC) of 11.34 (4–10 G/l) and natrium of 132 (136–146 mmol/l), blood tests were normal (lactate was not measured). The patient was transferred to our level-one trauma centre for surgical treatment. A secondary survey at admission did not expose any further injuries and the patient was moved to the ward with an anterior orthopaedic approach scheduled for the elective list after the weekend.

**Figure 1:**
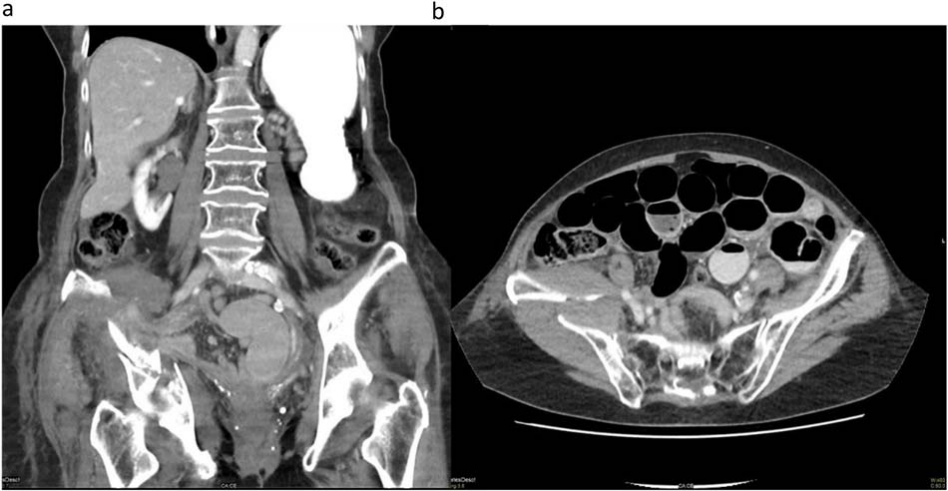
(a) Coronal CT image demonstrating the acetabulum fracture on the right side with herniated mesenteric fat tissue and collapsed small bowel loops (white arrow). (b) Transverse CT image demonstrating dilated small bowel loops and a distinct transition point (white arrow) on the right side close to the acetabulum fracture and a haematoma.

During hospitalization, the patient began vomiting, reported missing bowel movements and developed a distended abdomen with elevated bowel sounds. Blood tests indicated a decrease in haemoglobin to 117 (120–155 g/l) along with an elevated C-reactive protein (CRP) of 96.3 (<5 mg/l); lactate levels were not measured. A CT scan with intravenous contrast revealed a mechanical bowel obstruction with a distinct transition point from dilated to collapsed small bowel loops next to the fracture and a progressive haematoma. Radiologists initially misinterpreted the cause of the ileus, considering an increasing haematoma rather than herniated mesenteric fat with collapsed small bowel loops between parts of the acetabulum fracture as the potential cause (Fig. 1).

Subsequently, a nasogastric tube was placed, oral diet was restricted to liquids and parenteral fluid therapy was initiated. Although the patient remained clinically stable, the intestinal obstruction persisted, leading to the introduction of parenteral nutrition. Due to increasing lower abdominal pain and a CRP elevation to 130 (<5 mg/l), an exploratory laparoscopy was performed on the seventh day in the ward.

**Figure 2:**
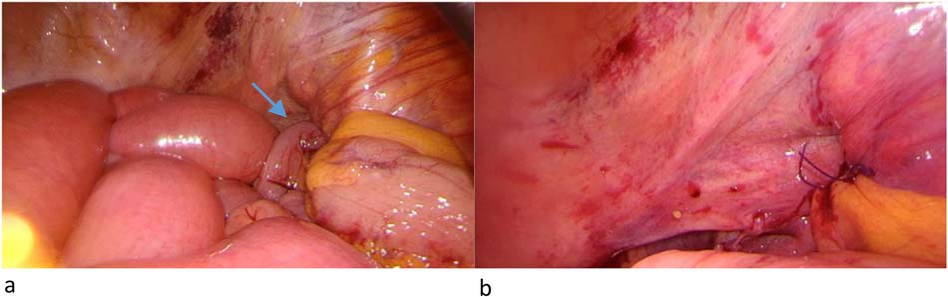
(a) Herniated loop (arrow) between the fractured fragment and (b) mesenterial fat patch.

During laparoscopy, distended small bowel loops, predominantly in the right lower abdomen accompanied by bloodstained ascites in all four quadrants, were present. An incomplete herniated loop was identified between the fractured fragments of the lamina quadrilateralis of the acetabulum on the right side. The myopectineal orifice was intact, without femoral or inguinal hernias. The herniated loop was freed from the defect, which measured about 20 × 5 mm. Minor oozing from the fracture was noted during the procedure. To create a tamponade and to avoid another herniation, we patched the hernia orifice with mesenterial fat from the ileocecal region with three resorbable intracorporal stitches (Fig. 2).

The postoperative course was uneventful, and the patient reported the first bowel movement on the 2nd postoperative day. The acetabulum osteosynthesis via an anterior approach was performed on day 5 after the laparoscopy, by which time the abdominal distention had resolved.

Transfer to rehabilitation was possible on the 10th postoperative day and the patient had an unremarkable follow-up 4 months later in the orthopaedic outpatient department.

## Discussion

The mechanisms involved in developing an acute TAWH are a combination of tangential forces and a sudden rise in intraabdominal pressure, disrupting the underlying muscle fibres, fascia and, eventually, the peritoneum. This type of hernia is mainly associated with motor vehicle accidents [6]. Sahdev’s criteria proposed in 1992 include the following characteristics to define a TAWH: no previous history of hernia, identification of the hernia after a traumatic event and a hernia sac may be present [7].

To date, there is no consensus on the diagnosis and treatment of traumatic abdominal hernias [8]. A CT scan is recommended, depending on the patient’s clinical picture, haemodynamic condition and associated fractures. We were only able to reach a definitive diagnosis by exploratory laparoscopy.

As initially suspected in our case, up to 40% of patients with pelvic and acetabular fractures develop a paralytic ileus, mainly due to retroperitoneal haematoma [9]. Nevertheless, mechanical obstruction can occur [4, 5] and has to be addressed accordingly. In the presented scenario, initially, a mechanical ileus due to a haematoma was suspected. Thus, a management plan, influenced by the Bologna criteria for the conservative treatment of mechanical bowel obstruction, was pursued [10]. With the knowledge gained from laparoscopy, a lower threshold for surgery may have been justified.

However, it is inevitable to assume that an early fracture treatment could have prevented the onset of the mechanical obstruction in the first place. On the other hand, an orthopaedic procedure via a possible posterior approach, before the laparoscopic evaluation, may have resulted in a bowel lesion with all the detrimental consequences for our patient.

## Conclusions

In summary, the management of TAWH remains challenging due to concomitant injuries and complications such as small bowel obstruction. Our case underscores the importance of good interdisciplinary communication, regular clinical examinations and re-evaluation of diagnostic results in patients who develop complications due to a TAWH or do not improve on treatment, respectively.

In cases of small bowel obstruction associated with pelvic fractures, a resolvable mechanical cause should be suspected, warranting operative treatment, even in the absence of a clear hernia orifice in cross-sectional imaging.

Lastly, we propose the inclusion of bony hernia orifices in future adaptations of the definition of TAWH.
